# Bortezomib-Induced Sensorineural Hearing Loss May Be Reversible with Intratympanic Dexamethasone

**DOI:** 10.3390/hematolrep18010009

**Published:** 2026-01-06

**Authors:** Natalia Peláez Casillas, Jose Maria Verdaguer Muñoz, Antonio Rodríguez Valiente, Irene Romera Martínez, Jose Ramón García Berrocal

**Affiliations:** 1 Servicio de Otorrinolaringología, Hospital Universitario Puerta de Hierro Majadahonda, Deparmento de Cirugía, Facultad de Medicina, Universidad Autónoma de Madrid, IDIPHISA, 28222 Majadahonda, Madrid, Spain; 2 Servicio Otorrinolaringología, Hospital Universitario HM Madrid Río, 28005 Madrid, Spain; 3 Servicio Hematología, Hospital Universitario Puerta de Hierro Majadahonda, 28222 Majadahonda, Madrid, Spain

**Keywords:** bortezomib, ototoxicity, sensorineural hearing loss, multiple myeloma, intratympanic dexamethasone

## Abstract

**Background**: Bortezomib, a proteasome inhibitor used in multiple myeloma (MM), is associated with several adverse effects, most notably peripheral neuropathy. Ototoxicity, however, remains a rare and underrecognized complication. **Case presentation**: We report the case of a 74-year-old man with MM who developed sudden unilateral sensorineural hearing loss following subcutaneous bortezomib administration. Audiometry confirmed severe right-sided hearing loss. MRI of the internal auditory canal was normal. Given the absence of other ototoxic agents, bortezomib was identified as the likely causative drug. The patient was treated with intratympanic dexamethasone injections, achieving partial hearing recovery. Subsequent chemotherapy re-exposure triggered another hearing decline, which again improved after repeated intratympanic treatment. **Conclusions**: Bortezomib-related ototoxicity is a rare but potentially reversible adverse event. This case suggests that early intratympanic corticosteroid therapy may mitigate cochlear injury, allowing continuation of chemotherapy for patients responding well to bortezomib.

## 1. Introduction

Multiple myeloma (MM) is a malignancy characterized by the clonal proliferation of plasma cells that infiltrate the bone marrow, leading to the production of monoclonal immunoglobulin (M-protein) and resulting in end-organ damage. This damage commonly manifests as hypercalcemia, renal insufficiency, anemia, and bone destruction—collectively known as the CRAB criteria [1]. MM is the second most common hematological malignancy in high-income countries, accounting for approximately 1–2% of all neoplastic diseases and 10–13% of all hematological malignancies globally [2].

Clinically, MM is associated with significant morbidity and mortality; at presentation, the most frequent findings include anemia (73%), osteolytic bone disease (79%), and acute kidney injury (19%) [3]. Although MM remains incurable, with most patients experiencing multiple relapses, overall survival has improved substantially due to the advent of modern therapeutic options, such as proteasome inhibitors, immunomodulatory drugs, monoclonal antibodies, autologous stem cell transplantation (ASCT) and chimeric antigen receptor (CAR) T-cell therapies. In recent years, the therapeutic landscape has further advanced with the introduction of BCMA-targeted and GPRC5D-targeted bispecific antibodies, which have shown remarkable efficacy in relapsed or refractory cases. Despite these advances, proteasome inhibitors continue to be a fundamental component of the standard treatment for MM.

Bortezomib, a first-generation proteasome inhibitor, revolutionized the treatment of multiple myeloma (MM) and related hematologic malignancies by targeting the 26S proteasome and disrupting protein degradation pathways [4]. Despite its proven efficacy, bortezomib may cause significant adverse effects, including peripheral neuropathy, thrombocytopenia, and gastrointestinal symptoms [5]. Ototoxicity, in contrast, is exceedingly rare and not well characterized in clinical practice. 

**Figure 1:**
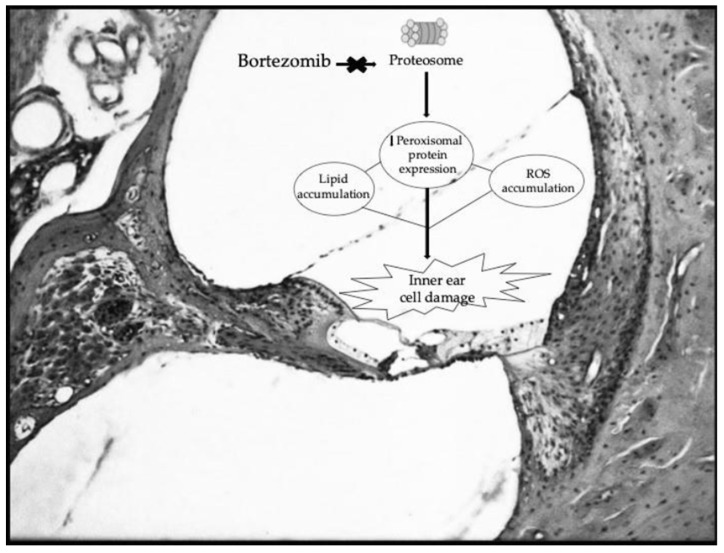
Schematic representation of the proposed bortezomib ototoxicity pathway adapted from experimental models [6]. The figure outlines the suggested mechanism by which bortezomib is hypothesized to contribute to cochlear toxicity, as evidenced in the present case. Bortezomib’s proteasome inhibition is proven to decrease peroxisomal protein expression. This reduction subsequently favors the accumulation of Reactive Oxygen Species (ROS) and lipids, leading to cellular damage within the cochlea.

The proteasome-ubiquitin system regulates numerous intracellular processes, including apoptosis, inflammation, and oxidative balance. Its inhibition has been shown to impair peroxisomal function, promoting reactive oxygen species (ROS) accumulation and auditory hair cell damage (Figure 1). Experimental studies have demonstrated that proteasome inhibitors can disorganize stereocilia bundles in the organ of Corti, resulting in irreversible hearing loss in animal models [6]. Following the initial report by Engelhardt et al. (2005) on irreversible deafness linked to bortezomib [7], only a few cases have been described in humans, most of them bilateral and permanent. To our knowledge, this is the first reported case of partial reversibility following intratympanic corticosteroid therapy.

## 2. Case Presentation

### 2.1. Hematological History and Initial Diagnosis

A 74-year-old Caucasian man was referred to the hematology department in February 2024 for severe asymptomatic neutropenia (absolute neutrophil count: 200/μL) and thrombocytopenia (111 × 10^9^/L) detected on a routine blood test one month after a mild SARS-CoV-2 infection. His past medical history included hypertension, dyslipidemia, and a prior episode of deep vein thrombosis and pulmonary embolism under chronic anticoagulation. No relevant family history was noted.

Further hematologic evaluation led to the diagnosis in March 2024 of IgA kappa multiple myeloma, standard risk (ISS Stage 1). Although he did not exhibit hypercalcemia, renal impairment, or anemia, at diagnosis the patient met the International Myeloma Working Group (IMWG) treatment criteria for active multiple myeloma [8], characterized by clonal bone marrow plasmacytosis >10% and evidence of organ damage, specifically a lytic lesion on PET-CT (fulfilling the ‘Bone’ criterion of the CRAB features).

Given his age, the patient was initially deemed ineligible for autologous stem cell transplantation (ASCT), but subsequent geriatric assessment confirmed he was clinically robust and suitable for intensive therapy. However, the patient declined to give his consent for the procedure, opting instead to proceed with standard induction therapy.

### 2.2. Multiple Myeloma Treatment Regimen

He initiated first-line chemotherapy in March 2024 with a daratumumab–bortezomib–dexamethasone (Dara-Vd). After three cycles, the patient achieved a partial hematologic response with good tolerance. From the fourth cycle, the regimen was intensified to Dara-VRD (adding lenalidomide). Shortly after the introduction of lenalidomide he developed a maculopapular rash on the arms and thighs. The regimen was eventually temporarily suspended due to severe neutropenia, requiring dose delays and G-CSF support. Between November and December 2024, he experienced several upper respiratory tract infections, however no antibiotics were administered, as the infections were viral in nature, ruling out antibiotic-induced ototoxicity.

Due to persistent cytopenias, the bortezomib dose was reduced to 1 mg/m^2^ (weekly subcutaneous administration) starting from the 8th cycle. From the 12th cycle onwards, the administration frequency was further reduced to biweekly due to hematological toxicity. Despite these dose adjustments and the safer subcutaneous route, the patient developed auditory symptoms during the 10th cycle, suggesting a high individual sensitivity or a cumulative toxic effect. On 27 February 2025, during the eleventh cycle, the patient was referred to the otolaryngology department with right-sided hearing loss.

### 2.3. Otological Presentation

The patient presented with a progressive 15-day history of right-sided hearing loss, not associated with tinnitus or vertigo. His otologic history included a right-ear barotrauma 11 years prior, benign paroxysmal positional vertigo of the right lateral semicircular canal (BPPV), alongside presbycusis. There was no history of chronic otitis media, or exposure to loud noise or other known ototoxic agents, as well as no family history of hearing loss.

Upon otological examination, otoscopy was unremarkable in both ears, and the cranial nerve examination, excluding the auditory component, was within normal limits. However, pure-tone audiometry (PTA) confirmed severe right-sided sensorineural hearing loss (SSNHL). MRI of the internal auditory canals revealed no structural abnormalities. Given the close temporal association between chemotherapy and auditory symptoms and noting that bortezomib is a documented but rare ototoxic agent while the other drugs in the regimen lack recognized ototoxic potential, bortezomib was identified as the probable causative agent. This initial episode of SSNHL was treated with intratympanic corticosteroids, leading to functional hearing recovery.

### 2.4. Results

**Figure 2:**
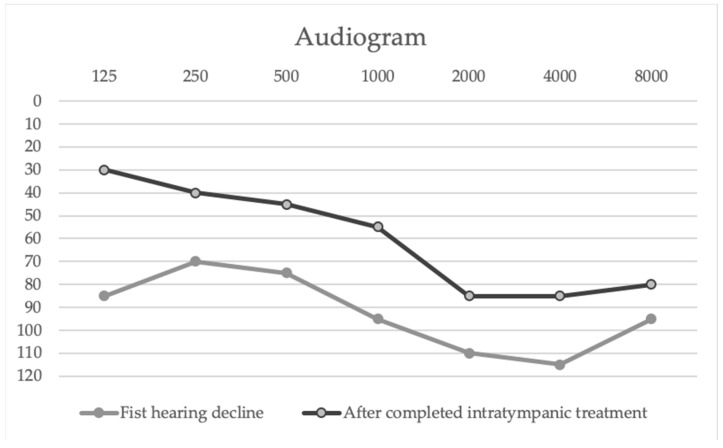
Audiogram showing the patient’s hearing thresholds after the first dose of bortezomib (First hearing decline) compared with those obtained following completion of intratympanic dexamethasone treatment (8 doses). The *X*-axis represents the frequencies in hertz (Hz), while the *Y*-axis shows the hearing level intensity in decibels (dB HL).

The patient received three intratympanic dexamethasone injections (4 mg/mL dexamethasone phosphate; 0.6–1 mL per dose, administered weekly) after the first hearing decline, with subjective and audiometric improvement. Following the next chemotherapy cycle, a second episode of hearing loss occurred; this was again treated with five intratympanic dexamethasone injections following the same weekly protocol and dosage, resulting in significant partial recovery (Table 1, Figure 2).

The left ear remained unaffected throughout treatment.

## 3. Discussion

Bortezomib-induced ototoxicity is a rare but clinically relevant complication, with only a handful of reported cases in the current literature. Prior reports have predominantly described severe, irreversible bilateral sensorineural hearing deficits, frequently necessitating the discontinuation of the crucial oncological treatment [7,9,10,11]. The underlying pathogenesis remains incompletely understood, yet current experimental models suggest peroxisomal dysfunction and oxidative stress within the cochlea as key contributors. Proteasome inhibition disrupts peroxisomal protein expression, impairs ROS detoxification, and induces lipid accumulation within cochlear hair cells, ultimately leading to stereocilia disorganization and apoptosis [6,12].

In our case, the auditory decline manifested during the tenth cycle of therapy. While previous literature often reports a sudden onset during the initial stages of treatment, this delayed presentation suggests that bortezomib-induced ototoxicity may also result from a cumulative dose-dependent mechanism. Although the lack of a baseline audiogram is a limitation, the strict temporal correlation between the tenth cycle and the symptomatic decline—in the absence of other ototoxic triggers—strongly supports bortezomib as the causative agent.

When comparing our case with previously reported irreversible instances [7,9,10,11], there are notable differences in the bortezomib administration schedule. While those cases often involved twice-weekly intravenous protocols, our patient received a weekly (and later biweekly) subcutaneous dose of 1 mg/m^2^. It remains unclear whether this less intensive schedule or the subcutaneous route played a role in the reversibility of the hearing loss, and further research is needed to determine if these factors influence auditory prognosis. However, in contrast to the irreversible outcomes previously documented in similar cases, our case uniquely demonstrates the partial reversibility of bortezomib-induced ototoxicity following local intervention. This successful outcome highlights the potential therapeutic role of timely local anti-inflammatory treatment using a weekly intratympanic dexamethasone protocol, which was a key factor in our patient’s clinical improvement.

Furthermore, the unilateral pattern observed is unusual. We hypothesize that the patient’s history of prior right-sided barotrauma may have increased the local vulnerability of the right cochlea, predisposing it to the systemic effects of bortezomib toxicity.

From a hematologic standpoint, continuation of bortezomib was recommended if possible, given the patient’s sustained clinical response, intolerance to lenalidomide and rejection of ASCT. Importantly, while the majority of similar cases have required the permanent cessation of bortezomib to prevent further damage, our patient was able to maintain this life-saving anti-myeloma regimen due to the functional hearing recovery achieved with focused therapy.

This successful outcome highlights the potential therapeutic role of timely local anti-inflammatory treatment. Corticosteroids exert multiple otoprotective mechanisms, including suppression of inflammatory cytokines (such as TNF-α and IL-1β), reduction in ROS accumulation, directly counteracting the proposed mechanism of bortezomib toxicity, and stabilization of ion homeostasis within the cochlea [13]. Given the need for high local drug concentration and minimal systemic effects in immunocompromised hematologic patients, intratympanic administration of corticosteroids achieves high perilymph concentrations safely and efficiently [14].

From a clinical standpoint, this case highlights several implications:

1. Baseline audiometric assessment before initiating bortezomib therapy may facilitate the early and accurate detection of ototoxicity.
2. Regular audiological monitoring could be recommended for patients, particularly those with pre-existing hearing impairment or otologic risk factors.
3. Prompt intratympanic corticosteroid therapy could promote partial reversibility and help maintain an effective chemotherapy regimen.
4. Multidisciplinary collaboration between hematologists and otolaryngologists is essential for early recognition and management of this uncommon auditory toxicity.

Further investigation is warranted to clarify the precise mechanisms of cochlear vulnerability, the optimal window for therapeutic intervention, and whether intratympanic corticosteroids could serve a definitive preventive or therapeutic role in chemotherapy-related ototoxicity.

## 4. Conclusions

This case report documents the first instance of significant partial hearing recovery from bortezomib-induced sensorineural hearing loss with intratympanic dexamethasone. This outcome stands in stark contrast to previous literature, which predominantly reported irreversible deficits often necessitating the cessation of this key anti-myeloma agent. The decision to maintain therapy under strict audiologic surveillance highlights the practical value of intratympanic corticosteroids as a localized, non-systemic intervention enabling oncologic treatment continuity in otherwise responding patients.

Clinicians should be aware of this rare complication and consider early otologic evaluation and corticosteroid therapy when auditory symptoms develop. Furthermore, while a rare complication, audiological monitoring should be considered in patients with pre-existing ear vulnerability or those reporting new onset symptoms during bortezomib therapy to ensure early detection and prevent the premature discontinuation of an effective systemic treatment for multiple myeloma.

## Figures and Tables

**Table 1:** Pure-Tone Liminal Hearing Thresholds. The table presents the pure-tone liminal hearing thresholds obtained via audiometry. The first row indicates the stimulus frequency values, expressed in kilohertz (kHz). Subsequent rows contain the numerical values of the hearing thresholds, expressed in decibels (dB HL), for the right ear (RE) and the left ear (LE).

	0.125	0.25	0.5	1	2	4	8	kHz
LE (control)	25	30	25	25	75	95	105	dB HL
First hearing decline (RE)	85	70	75	95	110	115	95	
After first intratympanic treatment (3 doses)	55	70	80	85	105	115	95	
Second hearing decline (RE)	55	65	75	75	105	115	105	
After second intratympanic treatment (5 doses)	35	50	40	50	90	100	100	
Latest Follow up (October 2025)	30	40	45	55	85	85	80	

## Data Availability

The data presented in this case report are available on request from the corresponding author. The data are not publicly available due to privacy or ethical restrictions.

## Abbreviations

The following abbreviations are used in this manuscript:

MM	Multiple myeloma
ROS	Reactive oxygen species
ASCT	Autologous stem cell transplantation
PTA	Pure tone audiometry
SSNHL	Sensorineural hearing loss
MRI	Magnetic resonance imaging
